# Effect of *MDR1 C3435T* and *CYP2C19* genetic polymorphisms on the outcome of *Helicobacter pylori* eradication treatment in children with gastritis and peptic ulcer, Vietnam

**DOI:** 10.1186/s12887-024-04581-w

**Published:** 2024-07-19

**Authors:** Loan Le Thi Thuy, Liem Thanh Nguyen, Hoang Anh Vu, Nghia An Nguyen, Tuan Anh Nguyen

**Affiliations:** 1 University of Medicine and Pharmacy at Ho Chi Minh City, Ho Chi Minh City, Vietnam; 2 Faculty of Nursing and Medical Technology, Can Tho University of Medicine and Pharmacy, Can Tho City, Vietnam; 3 Center for Molecular Biomedicine, University of Medicine and Pharmacy at Ho Chi Minh City, Ho Chi Minh City, Vietnam; 4 Department of Pediatrics, University of Medicine and Pharmacy at Ho Chi Minh City, Ho Chi Minh City, Vietnam; 5 Faculty of Medicine, Can Tho University of Medicine and Pharmacy, Can Tho City, Vietnam

**Keywords:** CYP2C19, MDR1 C3435T, Helicobacter pylori, Children

## Abstract

**Background:**

*Helicobacter pylori* eradication therapy based on antimicrobial susceptibility in Vietnamese children currently get low efficiency. There are causes of treatment failure, among host genetic factors namely *MDR1 C3435T* and *CYP2C19* affect the absorption and metabolism of proton pump inhibitors - a crucial component of eradication therapy. The study aimed to investigate the effect of *MDR1 C3435T* and *CYP2C19* genetic polymorphisms on the cure rate.

**Methods:**

207 pediatric patients with gastritis and peptic ulcer infecting *Helicobacter pylori* completed the eradication therapy based on antimicrobial susceptibility with proton pump inhibitor esomeprazole. Eradication efficacy was assessed after at least 4 weeks by the urease breath test. *MDR1 C3435T* genetic polymorphism and *CYP2C19* genotype were determined using a sequencing method based on Sanger’s principle.

**Results:**

Among 207 children recruited in this study, the ratio of CYP2C19 EM, IM, and PM phenotypes was 40.1%, 46.4%, and 16.9%, respectively. The patient with *MDR1 3435 C/C* polymorphism accounted for 43.0%, *MDR1 3435 C/T* was 40.1%, and *MDR1 3435T/T* was 16.9%. The cure rate of *Helicobacter pylori* infection in patients with CYP2C19 EM genotype was 78.3%; 83.3% of those with the IM genotype, and PM genotype was 96,4% (*p* = 0.07). Successful eradication rates for *Helicobacter pylori* were 85.4%, 86.7%, and 68.6% in patients with the *MDR1 3435 C/C*, *C/T*, and *T/T*, respectively (*p* = 0.02). Multiple logistic regression analysis found that *MDR1 C3435T* genetic polymorphisms of patients were significant independent risk factors for treatment failure, and *CYP2C19* genotype did not affect *Helicobacter pylori* eradication.

**Conclusions:**

The *Helicobacter pylori* eradication rates by regimens based on antibiotic susceptibility and esomeprazole were not significantly different between the CYP2C19 phenotypes. The *MDR1 C3435T* polymorphism is one of the factors impacting *Helicobacter pylori* eradication results in children.

## Background

*Helicobacter pylori (H. pylori)* infection is commonly acquired in children, resulting in chronic active gastritis. The treatment regimen for *H. pylori* eradication combines proton pump inhibitor drugs (PPIs), such as omeprazole, lansoprazole, rabeprazole or esomeprazole, and two antibiotic agents. The high treatment failure rate has posed a significant challenge for general physicians and gastroenterologists in recent years. Many factors might cause *H. pylori* treatment failure. Among the rapidly increasing and complicated development of antibiotic resistance in *H. pylori* and nonadherence to therapy are considered the important reasons [1]. In addition, host factors, including *CYP2C19* and *MDR1* genetic polymorphisms, may indirectly impact the efficacy of eradicating *H. pylori* because they alter the absorption and metabolism of PPIs.

PPIs are a key component of the *H. pylori* eradication regimen. They work by elevating the pH levels in the stomach, thereby increasing the stability and bioavailability of antibiotics within the stomach. In addition, PPIs also increase the sensitivity of the bacteria to antibiotics. The absorption of PPIs through oral administration is influenced by an ATP-dependent transporter protein namely P-glycoprotein (P-gp). The *MDR1* gene encodes P-gp. This gene has many polymorphisms and a polymorphic site at position 3435 in exon 26 has been evaluated to be related to the activity of P-gp [2]. Therefore, the concentration of PPIs in the serum may differ between *MDR1 C3435T* genotypes and may affect the therapy outcome for *H. pylori* infection. Recent research revealed that *MDR1 C3435T* polymorphism had an impact on the success or failure of *H. pylori* eradication by therapy based on PPIs including omeprazole, lansoprazole, and pantoprazole, and this impact is different among ethnicities on the world [3–5].

After PPIs are absorbed into the circulatory system, they are primarily metabolized in the liver by the enzyme CYP2C19. The *CYP2C19* gene is polymorphic, and encodes the enzyme S-mephenytoin 4’-hydroxylase, which metabolizes PPIs. The *CYP2C19* gene had 9 exons and 38 allele variants, which differ between the ethnic groups [6]. In the Vietnamese population, two mutations namely CYP2C19*2 in exon 5 and CYP2C19*3 in exon 4 are more popular than them in Caucasians, and CYP2C19*17 allele is rare [7]. Therefore, based on the degree of metabolism, three phenotypes are determined: extensive metabolizer (EM) with genotype CYP2C19*1/*1, intermediate metabolizer (IM) with genotypes CYP2C19*1/*2 and CYP2C19*1/*3, and poor metabolizer (PM) with genotype CYP2C19*2/*2, *2/*3, and *3/*3. The effectiveness of gastric acid inhibition by PPIs depends on the polymorphism of the *CYP2C19* gene and may affect the *H. pylori* treatment outcome. Some previous studies have found that the successful eradication rate in patients with poor or intermediate metabolizers was higher than in patients with extensive metabolizer [8]. The relationship between CYP2C19 phenotype and *H. pylori* eradication therapy effectiveness varies depending on the PPI drug, ethnicity, and *CYP2C19* polymorphism. Currently, there are many studies on these two genes in adults, but the results are conflicting, and the research on children is still limited. Therefore, we conducted this study to determine the impact of *CYP2C19* and *MDR1 C3435T* polymorphisms on the outcome of eradication therapy in Vietnamese children with gastritis and peptic ulcers to optimize the effectiveness of *H. pylori* eradication therapy in children.

## Materials and methods

### Study design and participants

An observational study was conducted at Can Tho Children’s Hospital and Can Tho University of Medicine and Pharmacy Hospital from March 2019 to April 2022. The sample size included 207 pediatric patients aged 6 to 16, who presented with gastrointestinal symptoms, and had esophagogastroduodenoscopy (EGD) indications. These patients had EGD and histologically proven peptic ulcer and chronic gastritis, positive *H. pylori* culture test, and carried out successful epsilometer test. Then the participants were treated *H. pylori* by regimen based on antimicrobial susceptibility with PPI was esomeprazole, monitoring, evaluating eradication outcome, and finally taking blood to identify genotyping of *CYP2C19* and *MDR1 C3435T* polymorphism.

Participants who were allergic to drugs in the regimen, did not follow the eradication protocol based on antimicrobial susceptibility results, had intolerances to therapy, or did not return to perform UBT for *H. pylori* outcome evaluation were excluded.

### EGD and biopsies

EGD was performed after the patient was completely anesthetized by well-trained endoscopists at the Endoscopy Center, University of Medicine and Pharmacy Hospital and Gastrointestinal Endoscopy unit, gastroenterology faculty, Can Tho Children’s Hospital. During upper endoscopy, we collected 04 gastric mucosa biopsies. One piece at the antrum and one piece at the body of the stomach were taken initially for *H. pylori* culture. One piece in the antrum was for the rapid urease test (NK Pylori test, Nam Khoa Biotek Co., Ltd), and one in the antrum for histopathology. The histopathological evaluation was applied to the updated Sydney classification [9].

### ***Culture and epsilometer test***

The culture was performed from the patient’s biopsies for whom the rapid urease test was positive. These biopsies are placed in a transportation medium and immediately transferred to the Department of Microbiology, Can Tho University of Medicine and Pharmacy for culturing. A single colony in culture medium for 4–5 days, determined based on colony morphology and the features of *H. pylori* including gram-negative-S shaped bacterium, urease positive, oxidase positive, and catalase positive. Finally, the antimicrobial susceptibility test was performed by epsilometer. Minimal inhibitory concentration (MIC) of 5 different antibiotics such as amoxicillin (AMX), clarithromycin (CLR), levofloxacin (LEV), tetracycline (TET), and metronidazole (MTZ) were determined by E-test (BioMerieux). According to the standards of the European Committee on Antimicrobial Susceptibility (EUCAST) 2019 to evaluate the susceptibility, the resistance cutoff values were 0.125 µg/mL for amoxicillin, 0.5 µg/mL for clarithromycin, 1 µg/mL for levofloxacin and tetracycline, 8 µg/mL for metronidazole [10].

### Tailored therapy and outcome evaluation

A 14 days regimen was tailored based on the results of the antimicrobial susceptibility test of the *H. pylori* strain isolated from gastric biopsies of every patient. It consisted of a PPI drug - was esomeprazole and two antibiotic agents to which *H. pylori* strain was susceptible for 14 days. In addition, pediatric patients with gastric ulcers were administered a standard dose of esomeprazole for 4 weeks, and 2 weeks for duodenal ulcer patients. Dosages of drugs were determined according to the 2017 ESPGHAN/NASPGHAN guidelines [11].

Following up and assessing the outcome of anti-*H. pylori* therapy: The eradicated results were judged at least 4 weeks after the tailored therapy was completed using the C^13^ or C^14^ urea breath test.

### Determination genotyping of CYP2C19 and MDR1 C3435T

Blood samples of patients were taken 2 mL of blood from the peripheral vein into a test tube with the anticoagulant EDTA, stored, and preserved at -80^0^C.

DNA extraction from a blood sample: genomic DNA was extracted from the peripheral blood using the GeneJET Whole Blood Genomic DNA Purification Mini Kit of Thermo Scientific according to the manufacturer^’^s protocol.

Genotyping was performed by the Sanger sequencing method to determine the nucleotide sequence of the CYP2C19*2 allele in exon 5, CYP2C19*3 allele in exon 4, and *MDR1 C3435T* polymorphisms in exon 26. The nucleotide sequences of the gene fragments were analyzed by ABI 3500 Genetic Analyzer (Applied Biosystem, USA).

The forward and reverse primer sequences of the CYP2C19*2 allele were designed as 5’- AACCAGAGCTTGGCATATTG-3’ and 5’- ATGTCCATCGATTCTTGGTG-3’, respectively. The primers that analyzed the CYP2C19*3 allele were 5’- TCTGCTCCATTATTTTCCAG − 3’ and 5’- TGGATTTCCCAGAAAAAAAG-3’, respectively. Subjects were classified into three groups: extensive metabolizers: EM (CYP2C19*1/*1), intermediate metabolizers: IM (CYP2C19*1/*2, CYP2C19*1/*3), poor metabolizers: PM (CYP2C19*2/*2, CYP2C19*3/*3, and CYP2C19*2/*3).

We directed gene sequencing of the *MDR1 C3435T* SNP, rs1045642 with primer sequence (Outer-MRD1-F: 5’- CCTGTTTGACTGCAGCATTG-3’and Outer-MDR1-R: 5’-GAAACATGACAGTTCCTCCAAGGCATAC-3’) was used to conduct the test. *MDR1 C3435T* genotypes were classified into three statuses, namely C/C, C/T, and T/T.

The experiments were carried out in the Center for Molecular Biomedicine, University of Medicine and Pharmacy at Ho Chi Minh City.

### Statistical analysis

Data were analyzed using the Statistical Package for Social Science (SPSS) version 20.0. Descriptive statistical analysis was used to describe the characteristics of the pediatric patients such as gender, age, gastroduodenal disease, susceptibility-resistance to antibiotics, outcome, EM/IM/PM phenotypes, and CC/CT/TT genotypes. The chi-square test was used to correlate the difference between proportions. Fisher’s exact test was used alternatively when more than 20% of the expected counts were less than 5. The logistic regression analysis determined the relationship between eradication outcomes and independent variables. A *p*-value less than 0.05 was accepted as statistical significance.

### Ethical issue

The study was conducted according to the guidelines of the Declaration of Helsinki and approved by the Ethics Committee of Ho Chi Minh University of Medicine and Pharmacy (approval No. 273/ĐHYD-HĐĐĐ dated 07 April 2019). Each patient obtained informed consent before participating in the study.

## Results

**Fig. 1:**
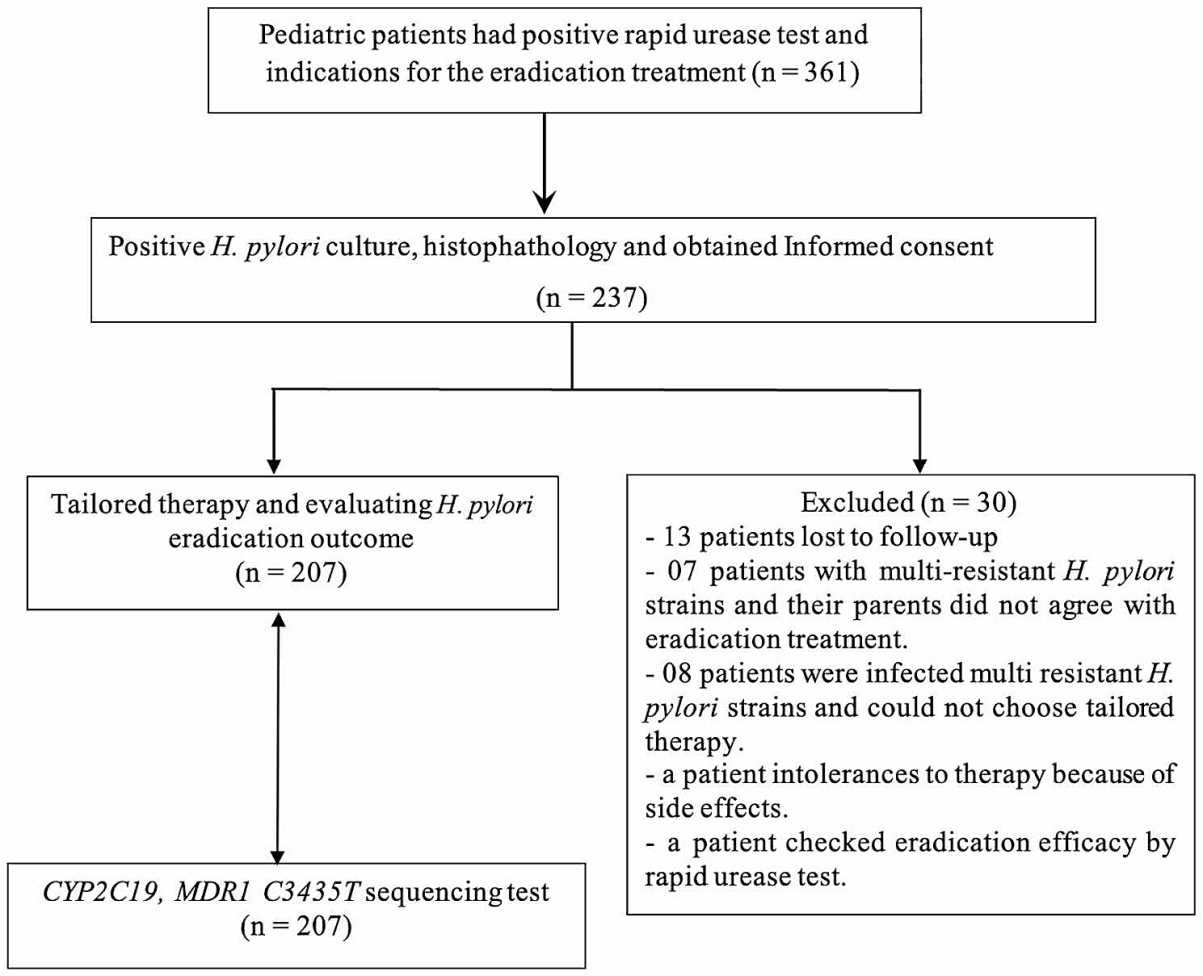
Flowchart of the study

We collected 361 pediatric patients assigned eradication treatment during the study period, and conducted *H. pylori* culture. There were 237 pediatric patients with positive *H. pylori* culture and successful antimicrobial susceptibility testing. Out of 237 pediatric patients enrolled in the study, 207 patients were eligible to undergo treatment and evaluate the efficacy of the regimen, and 30 patients were excluded from the analysis. Among excluded children, 13 patients lost to follow-up, EGD checked a patient, a patient was intolerant to therapy because of side effects, 07 patients were infected with multi-resistant strains of *H. pylori* and their parents did not agree with eradication treatment, 08 patients were not treated according to antimicrobial susceptibility results. Finally, we conducted sequencing *CYP2C19* and *MDR1 C3435T* genomic for the returned patients (Fig. 1).

### Characteristics of patients

Among 207 pediatric patients in the study, we showed that the percentage of males was 48.8% (*n* = 101); the mean age was 10.7 ± 2.4 years old. Patients with gastritis were 69.6% (*n* = 144), the duodenal ulcer was 28.0% (*n* = 58), and the gastric ulcer was 2.4% (*n* = 5). The patients with mild *H. pylori* bacterial load accounted for 62.8% (*n* = 130), moderate was 17.4% (*n* = 36), and severe was 19.8% (*n* = 41). Resistance rates of *H. pylori* were 79.2% to clarithromycin, 70.5% to amoxicillin, 43.5% to metronidazole, 44.4% to levofloxacin, and 10.6% to tetracycline. The successful *H. pylori* eradication rate of the tailored therapy base on the 2017-ESPGHAN/NASPGHAN guideline was 83.1% (172/207).

The proportion of the CYP2C19*2, and *3 alleles accounted for 29.7% and 6.8%, respectively; there was no significant difference in the proportions of the CYP2C19*2 and *3 alleles between male and female patients. The frequencies of CYP2C19 EM, IM, and PM phenotypes in the study were 40.1%, 46.4%, and 13.5%, respectively. The distribution of CYP2C19 EM, IM, and PM phenotype in the gastritis group was not significantly different from the peptic ulcer patient group (*p* > 0.05) (Table 1).

**Table 1:** The CYP2C19 polymorphisms in *H. pylori*-infected pediatric patients

CYP2C19 genotype	n (%)	CYP2C19 phenotype	n (%)	EGD Diagnosis	
				Gastritis	Peptic ulcer
*1/*1	83 (40.1)	**EM**	83 (40.1)	58 (40.3)	25 (39.7)
*1/*2	81 (39.2)	**IM**	96 (46.4)	67 (46.5)	29 (46.0)
*1/*3	15 (7.2)				
*2/*2	18 (8.7)	**PM**	28 (13.5)	19 (13.2)	9 (14.3)
*2/*3	5 (2.4)				
*3/*3	5 (2.4)				
**Total**	**207 (100)**		**207 (100)**	**144**	**63**

*EM, Extensive metabolizer; IM, Intermediated metabolizer; PM, Poor metabolizer*

About the *MDR1 C3435T* polymorphism, the proportion of the *MDR1 3435T* allele was 37.0% lower than that of the *3435 C* allele, at 63.0%. In a total of 207 patients, 43.0% (89/207) were *MDR1 3435 C/C* genotype, 40.1% (83/207) were *C/T* genotype, and 16.9% (35/207) were *T/T* genotype. There was no significant difference in the *MDR1 C3435T* genotype distribution between gastritis to peptic ulcer patient group (Table 2).

**Table 2:** The *MDR1 C3435T* polymorphisms in *H. pylori*-infected pediatric patients

MDR1 C3435T	n (%)	EGD Diagnosis		*p*-value
		Gastritis	Peptic ulcer	
**Allele**				
***3435 C*** ***3435T***	261 (63.0) 153 (37.0)	179 (62.2) 109 (37.8)	82 (65.1) 44 (34.9)	0.6
***MDR1 C3435T*** **genotype**				
***C/C***	89 (43.0)	60 (41.7)	29 (46.0)	0.6
***C/T***	83 (40.1)	59 (41.0)	24 (38.1)	0.7
***T/T***	35 (16.9)	25 (17.4)	10 (15.9)	0.8

*MDR1, Multidrug resistance.*

### Factors associated with the *H. pylori* tailored therapy outcome

Three factors significantly influenced to cure rate: age, EGD diagnosis, and *MDR1 C3435T* genotype. There was no statistically significant difference in the outcome of *H. pylori* therapy by gender, history of previous *H. pylori* treatment, the load of *H. pylori* on the histopathology test, and *CYP2C19* genotype (*p* > 0.05) (Table 3).

**Table 3:** Univariate analysis of factors influencing *Helicobacter pylori* eradication rate of tailored therapy

Factor	n	Eradication outcome		*p*-value	OR (95% CI)
		Success	Failure		
**Mean age (year)** **Aged group**	10.7 ± 2.4	11.4 ± 2.29	9.0 ± 2.36	*< 0.001*	
**6–10 years old** **11–16 years old**	98 109	70 (71.4) 102 (93.6)	28 (28.6) 7 (6.4)	***< 0.001***	5.83 (2.41–14.09)
**Gender**					
Male Female	101 106	84 (83.2) 88 (83.0)	17 (16.8) 18 (17.0)	0.98	0.99 (0.48–2.05)
**EGD diagnosis**				***0.03***	3.05 (1.13–8.28)
Gastritis Peptic ulcer	144 63	114 (79.2) 58 (92.1)	30 (20.8) 5 (7.9)		
***H. pylori*** **load**					
Mild Moderate and severe	130 77	112 (86.2) 60 (77.9)	18 (13.8) 17 (22.1)	0.18	1.76 (0.85–3.67)
***CYP2C19*** **genotype**					
EM IM PM	83 96 28	65 (78.3) 80 (83.3) 27 (96.4)	18 (21.7) 16 (16.7) 1 (3.6)	0.19 1.0 0.06^a^	1.74 (0.84–3.62) 1.03 (0.5–2.14) 5.32 (0.76–37.3)
***MDR1 C3435T***					
C/C C/T T/T	89 83 35	76 (85.4) 72 (86.7) 24 (68.6)	13 (14.6) 11 (13.3) 11 (31.4)	0.46 0.27 ***0.02*** ^*^	1.34 (0.63–2.83) 1.57 (0.72–3.41) 2.83 (1.23–6.51)

*(*^*a*^*): Fisher*^*’*^s Exact Test, (^*^): p = *0.02 compared with C/C and C/T.*

*EM, Extensive metabolizer; IM, Intermediated metabolizer; PM, Poor metabolizer.*

The lowest cure rate was 78.3% for pediatric patients with CYP2C19 EM phenotype, 83.3% for CYP2C19 IM, and the highest of them was 96.4% for CYP2C19 PM, but no significant differences in the eradication rates between different CYP2C19 phenotypes. In the *MDR1 C3435T* polymorphism analysis, the cure rates for *MDR1 3435T/T* were lowest with 68,6%, *C/T*, and *T/T* genotypes were 85.4%, and 86.7%, respectively (*p* = 0.02). The patients with *MDR1 3435T/T* genotype were 3 times more likely to fail treatment than *MDR1 3435 C/C* and *C/T*.

The patients aged 6 to 10 years old had a reasonably lower cure rate than those those aged 11 to 16 years old (71.4 vs. 93.6, OR = 5.83, 95% CI = 2.41–14.09, *p* < 0.001).

Table 4 showed the summary of *CYP2C19* genotype and *MDR1 C3435T* polymorphism with the therapy outcome. The pediatric patients with the simultaneous CYP2C19 EM phenotype and *MDR1 3435T/T* had the lowest cure rate of *H. pylori* infection, only at 60%. In contrast, the patients carrying both CYP2C19 PM phenotype and *MDR1 3435 C/C or C/T* had the highest successful eradication rate, getting at 100%, and this patient group has an increased chance of successful eradication therapy compared to those with CYP2C19 EM or IM phenotypes and *MDR1 3435T/T* (*p* = 0.02).

**Table 4:** Summary of the *CYP2C19* genotype and *MDR1 C3435T* polymorphism with therapy outcome

CYP2C19 genotype	MDR1 C3435T	Successful eradication	Failure eradication	*p*-value
EM (*n* = 83)	C/C (*n* = 34) C/T (*n* = 39) T/T (*n* = 10)	27 (79.4) 32 (81.2) 6 (60.0)	7 (20.6) 7 (17.8) 4 (40.0)	0.53 1.0 0.07^*^
IM (*n* = 96)	C/C (*n* = 42) C/T (*n* = 34) T/T (*n* = 20)	36 (85.7) 30 (88.2) 14 (70.0)	6 (14.3) 4 (11.8) 6 (30.0)	0.7 0.5 0.1
PM (*n* = 28)	C/C or C/T (*n* = 23) T/T (*n* = 5)	23 (100.0) 4 (80.0)	0 (0.0) 1 (20.0)	***0.02*** ^*^ 1.0

*(* ^*^ *)Fisher* ^*’*^ *s Exact Test*

Effect of the *CYP2C19* genotype and *MDR1 C3435T* polymorphism on the *H. pylori* eradication rates. We performed a multivariable logistic regression analysis of factors impacting the treatment outcome, including age, EGD diagnosis, *CYP2C19* genotype, and *MDR1 C3435T* polymorphisms. The results suggested that age and *MDR1 C3435T* had related to the cure rate of *H. pylori* (Table 5).

**Table 5:** Multivariate logistic regression analysis of factors that affected the outcomes of anti-*H. pylori* treatment

Factor	OR	95% Cl	*p*
**Age**	1.47	1.21–1.79	***< 0.001***
**EGD diagnosis**			
Gastritis Peptic ulcer (Ref)	2.28 1.00	0.76–6.83	0.14
**CYP2C19 phenotype**			
EM (Ref) IM PM	1.00 2.03 8.07	0.86–4.75 0.96–67.97	0.10 0.06
***MDR1 C3435T***			
C/C C/T T/T (Ref)	3.74 3.63 1.00	1.25–11.22 1.26–10.48	***0.02*** ***0.02***

## Discussion

### Characteristics of patients

Our research showed that the frequencies of CYP2C19 EM, IM, and PM phenotypes in pediatric patients infected with *H. pylori* with gastritis and peptic ulcer were 40.1%, 46.4%, and 13.5%, respectively. We noticed differences in the distribution of CYP2C19 phenotype between the research in Vietnamese populations. The CYP2C19 PM prevalence was reported from 6.3 to 9.0% in the previous study, and 13.5% in the Mekong Delta, Vietnam (our study). The frequencies of CYP2C19 phenotypes in our study are similar to the reports of Asian countries, such as China - Zhang Y. in 2020 (CYP2C19 EM was 47%, CYP2C19 IM was 44%, and CYP2C19 PM was 10%) [12]; Japan - Okimoto T. in 2016 (CYP2C19 EM was 32.4%, IM was 47.0%, and PM was 20.5%) [13]; and Thailand - Auttajaroon J. in 2019 (CYP2C19 EM was 55.9%, IM was 40.9%, and PM was 3.2%) [14]. However, these results were different compared to the reports in the Caucasians as Bernal C.J. in 2019 in the United States (CYP2C19 UM was 33%, CYP2C19 EM was 40%, and PM/IM was 27%) [15], Omeci in 2016 in Turkey (CYP2C19 EM was 78.0%, IM was 19.5%, and PM was 2.5%) [16]. In general, most pediatric patients with indications for *H. pylori* eradication treatment have the CYP2C19 EM and IM phenotypes. Thus, our results suggest that if the *CYP2C19* genotype is unknown, clinicians should not choose the first generation PPIs, such as omeprazole, lansoprazole, or pantoprazole on the anti-*H. pylori* therapy in Vietnamese children.

The proportion of the *MDR1 3435T* allele was 37.0% lower than that of the *MDR1 3435 C* allele, at 63.0%. The distribution of the *MDR1 C3435T* genotypes is the same as the previous research in Asia such as: Oh J.H. in Korea (*MDR1 3435 C/C* was 35.7%, *MDR1 3435 C/T* was 51.9%, and *MDR1 3435T/T* was 12.4%) [17], Tahara T. in Japan (*MDR1 3435 C/C* was 36.9%, *MDR1 3435 C/T* was 45.7%, and *MDR1 3435T/T* was 17.4%) [18], and BaniHani N.M. in Jordan (*MDR1 3435 C/C* was 27.3%, *MDR1 3435 C/T* was 52.1%, and *MDR1 3435T/T* was 20.6%) [19]. However, our findings demonstrate a lower percentage of the *MDR1 3435T/T* compared to the studies conducted in Europe and Australia such as Salagacka A. in Poland (*MDR1 3435T/T* was 34.2%) [20], Karaca R.O. in Turkey (*MDR1 3435T/T* was 25.4%) [5], and Omar M. in Australia (*MDR1 3435T/T* was 30.3%) [21]. This polymorphism distribution generally differ from the research in other parts of the world. This can be explained by polymorphisms related to inherited traits, so they vary between ethnicities. Our study on the different distribution of the *MDR1 C3435T* polymorphism in pediatric patients with gastritis and peptic ulcers may be useful in modifying drug selection on personalized therapy. The PPIs, a substrate of P-pg, play an important role in the anti - *H. pylori* treatment. Patients with a high level of P-gp activity are at risk of failure of eradication therapy due to the poorly absorbed PPI drugs into intestinal epithelial cells. Therefore, it would be beneficial to recognize the tissue of the *MDR1 C3435T* genotype distribution, from that we can do the personalized eradication therapy and get a higher eradication outcome.

### Factors impact the outcome of *Helicobacter pylori* treatment

#### The relationship between CYP2C19 phenotype and the effectiveness of *Helicobacter pylori* treatment

According to the ESPGHAN/ NAPGHAN 2017 guideline, in our study, the PPI drug utilized in the anti-*H. pylori* treatment was esomeprazole, the cure rate in pediatric patients with CYP2C19 EM phenotype was 78.3%, IM was 83.3%, and PM was 96.4%. There was no difference in the *H. pylori* eradication rates between the CYP2C19 phenotypes (*p* > 0.05). Several studies worldwide have shown the influence of the *CYP2C19* polymorphism on *H. pylori* eradication treatment outcomes with esomeprazole. In Asia, the research of Okimoto T. et al. (2016) Japan, 108 patients treated for seven days with EAC regimens (esomeprazole, amoxicillin, and clarithromycin), the success rate in the CYP2C19 EM phenotype was 77.3%, the IM phenotype was 75.5%, and the PM phenotype was 71.4%; the CYP2C19 phenotype had no effect on treatment outcome [13]. Similarly, the study of Su J. in China in 270 *H. pylori*-infected patients treated by the triple regimen with PPI was esomeprazole, the success rate of *H. pylori* treatment in the CYP2C19 EM and IM phenotypes was 84.0%, and PM was 84.4%, the *CYP2C19* polymorphism did not affect the outcome of *H. pylori* treatment [22]. In Europe, a study conducted by Miehlke S. in Germany on 103 patients who had previously failed anti-*H. pylori* therapy showed that the eradication rate was higher among patients with CYP2C19 PM and IM phenotypes (93.1%) compared to the EM phenotype (78.8%) (*p* = 0.059) [23]. Our results were consistent with the above studies. When using 3 or 4 drug regimens with the PPI drug esomeprazole, the *CYP2C19* polymorphism did not affect the results of *H. pylori* treatment. However, this is unconsistent with some studies, such as the research of Kuo C.H. in 2010 in Korea [24], Saito Y. in 2015 in Japan [25]. These studies found that the *H. pylori* cure rate was significantly differed between the CYP2C19 phenotypes.

With the current data source, we did not find a study on the effect of *CYP2C19* genetic polymorphism on the efficacy of *H. pylori* eradication therapy by the regimen consisting of esomeprazole in children. Previous studies have rarely mentioned the influence of *CYP2C19* genetics on *H. pylori* eradication in children. In 2010, a review of Chiesa C. only discussed the factors influencing therapy outcomes, including antibiotic resistance, adherence, and bacteria factors [26]. The study of Settin A. in Egypt in 2014 on 100 gastritis pediatric patients who were eradicated by the triple regimen of lansoprazole for 14 days. The cure rate of the patient group with the CYP2C19 IM and PM phenotypes was higher than in the EM phenotype group (84.6% and 77.8% vs. 69.2%), and this difference is not statistically significant [27]. The weakness of the study is the lack of data on antibiotic resistance of *H. pylori* and the small sample size, the CYP2C19 PM phenotype group has 9 cases, of which 7 were successful, and 2 fail. In 2020, a research by Zhang Y.D. in China on 156 pediatric patients infected with *H. pylori* who failed with a standard triple regimen (omeprazole, amoxicillin and clarithromycin). The participants were divided into two groups. Group 1 included 64 children, who did not perform *H. pylori* culture and CYP2C19 gene test, eradicated treatment by bismuth, esomeprazole, amoxicillin, and metronidazole regime, the cure rate reached 72%. Group 2 included 92 children, the eradication rate got 99% in pediatric patients who were treated based on antimicrobial susceptibility and CYP2C19 phenotype, and 88% in the patients whose *H. pylori* culture failed, treatment only based on CYP2C19 phenotype. The study advised that *H. pylori* treatment in children should be based on antimicrobial susceptibility and *CYP2C19* genotype [12].

According to the literature, there are many factors affecting the effectiveness of the *H. pylori* eradication treatment, among which the important factors are the antibiotic resistance of bacteria and adherence to therapy of the patient. Some studies about the effect of *CYP2C19* genotype on the outcome of *H. pylori* eradication therapy did not address these two basic factors and maybe this is one of the reasons leading to the difference in the research results. The strength of our study is the anti-*H. pylori* therapy based on the guideline of ESPGHAN/NAPGHAN 2017 and excluded cases of intolerance to therapy. Although the difference in the cure rates between the CYP2C19 phenotype groups was not statistically significant, we found that the eradication rate was higher in the CYP2C19 PM phenotype patients than the CYP2C19 EM and IM phenotypes. With the development of personalized medicine, the effectiveness of *H. pylori* therapy can be further improved when treatment is based on the *CYP2C19* genotype.

#### The relationship between the MDR1 C3435T genotypes and the effectiveness of Helicobacter pylori treatment

The study found that the *H. pylori* cure rate in pediatric patients with *MDR1 3435T/T* genotype was lower than that in *MDR1 3435 C/C* and *3435 C/T* genotypes. The difference in *H. pylori* eradication rate between 3 genotype groups, namely *MDR1 3435T/T, C/T*, and *T/T* was statistically significant. Similar to the studies in Asian countries such as Japan, and Korea about the influence of the *MDR1 C3435T* polymorphism on the effectiveness of the *H. pylori* eradication treatment. A previous study by Furuta T. et al. in Japan, in 2007 conducted on 313 adult patients with gastritis and peptic ulcer, found that the success rate of the *H. pylori* treatment in the patient group with *MDR1 3435T/T* genotype was 67%, which was statistically significantly lower than in *MDR1 3435 C/C* and *3435 C/T* genotypes were 82% and 81%, respectively (*p* = 0.004) [3]. The research by Oh J.H. et al. in 2009 in Korea, eradicated *H. pylori* in 210 patients with gastritis and peptic ulcer by the triple regimen based on pantoprazole, amoxicillin and clarithromycin for 7 days. The successful eradication rate of the patient group with the *MDR1 3435T/T* genotype was 76.9%, lower than that in the *MDR1 3435 C/C* and *3435 C/T* genotypes, 82.7% and 84.4% respectively. However, the difference in *H. pylori* cure rate between genotype groups *MDR1 3435 C/C*, *3435 C/T* and *3435T/T* was not statistically significant (*p* > 0.059) [17]. A review analysis by Li M. in 2017 also concluded the same, the *MDR1 3435T/T* genotype was associated with low eradication rates in patients receiving triple regimens with omeprazole or lansoprazole [28]. Therefore, the *MDR1 C3435T* polymorphism affects the outcome of the *H. pylori* eradication treatment by the regimens with PPIs, and the *MDR1* 3435*T/T* genotype reduces the *H. pylori* eradication rate in Asians.

However, similar research was done by Gawronska-Szklarz B. et al. in Poland and Karaca O.R. et al. in Turkey showed that the successful eradication rate of *H. pylori* in patients with the *MDR1 3435T/T* genotype was higher than the *MDR1 3435 C/C* and *C/T* genotypes, and found that the *H. pylori* cure rate between the *MDR1 C3435T* genotypes did not differ statistically significantly [4, 5]. It is opposite to our result. The basic research of Hoffmeyer et al. in Germany reported that the single nucleotide polymorphism at position 3435 on exon 26 was associated with the activity level of P-pg in the small intestine. The subjects having a homozygous T allele were associated with a 2-fold lower expression level of the *MDR1* gene than those with a homozygous C allele [2]. Thus, the plasma level of drugs, which is the substrate of P-gp and is used orally, was higher in carriers of the *MDR1 3435T/T* genotype than those of *MDR1 3435 C/C.* Resulting in a higher *H. pylori* cure rate among patients with *MDR1 3435T/T* genotype compared to those with the *MDR1 3435 C/C* and *C/T* genotype in the Caucasian population. This explains the different impact of the *MDR1 C3435T* polymorphism on the efficacy of *H. pylori* eradication treatment using PPI-based regimens between Asians and Caucasians.

#### Some factors affect the outcome of Helicobacter pylori eradication treatment in children

Through our analysis, we demonstrated that some factors that can affect the outcome of *H. pylori* eradication treatment by tailored therapy based on PPI are esomeprazole in pediatric patients with gastritis and peptic ulcer including age, gastroduodenal disease diagnosis, CYP2C19 phenotypes, and *MDR1 C3435T* genotype. We carried out multivariate regression analysis to remove confounding factors. The results showed that age and *MDR1 C3435T* polymorphism are two independent factors affecting the effectiveness of *H. pylori* eradication therapy.

Regarding the aged factor of pediatric patients, the study found that the *H. pylori* cure rate in the 11–16 aged group was statistically significantly higher than those in the 5–10 aged group, and if the patient increases plus one year old, *H. pylori* successful eradication treatment ability will increase 1.47 times (95% CI: 1.21–1.79, *p* < 0.05). Today, very few studies consider age as the factor affecting the effectiveness of eradication treatment. According to the Japanese Society for Pediatric Gastroenterology, Hepatology and Nutrition guideline in 2020, it is recommended to consider *H. pylori* eradication therapy in children 5 years of age and older [29]. However, the present study recommended that we consider treating *H. pylori* eradication for children younger than 11 years old. Hence, it is essential to conduct well-designed studies with a large sample size to investigate this factor that could potentially impact the effectiveness of *H. pylori* eradication in children.

The study found that the host genetic factor, namely *MDR1 C3435T* polymorphism is an independent factor affecting the effectiveness of *H. pylori* eradication treatment in children. With the development of clinical pharmacogenetics, the previous treatment model, namely one size/one drug fit all is no longer relevant. The study of Kodaira C. et al. demonstrated that the MDR1 C3435T polymorphism influenced the pharmacokinetics, but not the pharmacodynamics (i.e., intragastric pH), of lansoprazole in rapid metabolizers of CYP2C19 [30]. Therefore, We recommend that study about the association with pharmacokinetics (plasma level of esomeprazole) and *MDR1 C3435T* genetic polymorphisms in Vietnamese pediatric patients should be conducted in the future.

The *CYP2C19* polymorphism does not affect the efficacy of *H. pylori* eradication therapy by regimen based on antimicrobial susceptibility with the PPI drug esomeprazole. Therefore, if the anti-*H. pylori* treatment based on antimicrobial susceptibility and using a PPI drug is esomeprazole, the routine *CYP2C19* genetic test is not necessary to choose a specific regimen for subjects with CYP2C19 EM improved the eradication rate in children.

## Conclusion

The *H. pylori* eradication rates by regimen based on antimicrobial susceptibility with esomeprazole were not significantly different between the CYP2C19 phenotypes. The *MDR1 C3435T* polymorphism is one of the factors impacting the *H. pylori* eradication outcome in children.

## Data Availability

The data is available from the corresponding author upon reasonable request.

## Abbreviations

*H. pylori*: *Helicobacter pylori*
PPI: Proton pump inhibitor
